# Co-designing an interactive artificial intelligent system with post-stroke patients and caregivers to augment the lost abilities and improve their quality of life: a human-centric approach

**DOI:** 10.3389/fpubh.2023.1227748

**Published:** 2023-09-21

**Authors:** Sara Ventura, Giovanni Ottoboni, Giada Lullini, Rabih Chattat, Laura Simoncini, Elisabetta Magni, Roberto Piperno, Fabio La Porta, Alessia Tessari

**Affiliations:** ^1^Department of Psychology, University of Bologna, Bologna, Italy; ^2^Instituto Polibienestar, University of Valencia, Valencia, Spain; ^3^IRCCS Istituto delle Scienze Neurologiche di Bologna, Bologna, Italy; ^4^Montecatone Rehabilitation Institute, Imola, Italy; ^5^Alma Mater Research Institute for Human-Centered Artificial Intelligence, University of Bologna, Bologna, Italy

**Keywords:** intelligent assistive technology, stroke patients, caregivers, technology acceptance, human-centric design

## Abstract

**Objectives:**

The motor disability due to stroke compromises the autonomy of patients and caregivers. To support autonomy and other personal and social needs, trustworthy, multifunctional, adaptive, and interactive assistive devices represent optimal solutions. To fulfill this aim, an artificial intelligence system named MAIA would aim to interpret users’ intentions and translate them into actions performed by assistive devices. Analyzing their perspectives is essential to develop the MAIA system operating in harmony with patients’ and caregivers’ needs as much as possible.

**Methods:**

Post-stroke patients and caregivers were interviewed to explore the impact of motor disability on their lives, previous experiences with assistive technologies, opinions, and attitudes about MAIA and their needs. Interview transcripts were analyzed using inductive thematic analysis.

**Results:**

Sixteen interviews were conducted with 12 post-stroke patients and four caregivers. Three themes emerged: (1) Needs to be satisfied, (2) MAIA technology acceptance, and (3) Perceived trustfulness. Overall, patients are seeking rehabilitative technology, contrary to caregivers needing assistive technology to help them daily. An easy-to-use and ergonomic technology is preferable. However, a few participants trust a system based on artificial intelligence.

**Conclusion:**

An interactive artificial intelligence technology could help post-stroke patients and their caregivers to restore motor autonomy. The insights from participants to develop the system depends on their motor ability and the role of patients or caregiver. Although technology grows exponentially, more efforts are needed to strengthen people’s trust in advanced technology.

## 1. Introduction

Stroke is the most prevalent cause of motor disability (1). Around 15 million people are estimated to suffer a stroke yearly, and almost 33% have a permanent disability (2, 3). Cognitive, emotional, and sensory disorders often emerge after a stroke; upper and lower extremity weakness or hemiparesis is the most common impairment (4). After discharge from the hospital, many patients may lose their autonomy in daily life. They struggle to return to work, suffer from significant social restrictions, and have decreased quality of life (5, 6).

Stroke also firmly impacts informal caregivers—generally, spouses or adult children—(7–9), who provide most of the stroke survivor’s daily care (10). Previous studies demonstrated that unexpected life changes, the related strain, and the worry about the care recipient’s health cause anxiety, depression, and decreased quality of life in most caregivers (11). On the other hand, although improvements in acute and long-term medical care increase life expectancy (12), they also require prolonged support.

In this light, advanced technologies, including Assistive Technologies (ATs), may be crucial for supporting people with disabilities and their caregivers (13, 14). ATs refer to “any product or technology-based service that enables people of all ages with activity limitations in their daily life, education, work or leisure” (15). They span across the software to support and train cognitive functions or to support the caregiver in the care management activities (16, 17), toward pieces of hardware—such as exoskeleton—to compensate for motor function and support daily activities (18, 19), or are embodied by social robots supporting caregivers in heavy tasks (20). In this line, ATs could be considered Human Augmentation as they enhance human abilities (21). Modern advancements in science and technology have led to a great variety of brain implants that permit, through Artificial Intelligence (AI) systems, to integrate information detected by the user and to adapt computer input to match the user’s situational needs. Therefore, a closed loop is formed between the user and the technological interface. This advanced AT can assist and autonomously support a variety of tasks that users are unable or unwilling to perform after a stroke (22). However, according to the literature, too many ATs for people with disabilities are still developed without considering the real needs of the final users with the risk of abandonment by their use or the lack of interest (23, 24).

The user-centric design method seems promising because it includes users’ perspectives during the design of the technology and leads to a device’s development that better suits users’ needs (25). Through the human-centric method, the end-users are involved from the earliest stages of the technology design by expressing evaluations and opinions about the proposed system’s acceptability, usability, ease of use, usefulness, and appropriateness. End-user involvement includes the collection of insights and testimonies concerning realistic problems and obstacles, whose analysis and solution increase technology acceptability and compliance (26). According to the Technology Acceptance Model (TAM), the more the technologies fit users’ needs, the more they are accepted (27–29). Thus, one of the leading technological improvements regards the interexchange between the end-user and the device. The feature to control the device via thought analysis would represent a robust solution in many cases (30).

To fulfill this goal, the European project MAIA (Multifunctional Adaptive and Interactive Artificial Intelligent system for acting in multiple contexts; Grant agreement ID: 951910) is developing AI-guided interfaces to control devices such as robotic arms, electronic wheelchairs, or exoskeletons. MAIA is designing a sensor implanted into the parietal cortex to revive autonomy in post-stroke patients. The sensor will extract the neural signals generated during the imagery of reaching and grasping hand gestures from the posterior parietal cortex (31). The sensory and motor information will be appropriately decoded via AI to effectively lead an external device’s movements. The development of MAIA begins from the end-users’ perspective. Therefore, an essential aspect of the project is the aforementioned human-centric approach.

In the present article, we report the qualitative exploration of the general point of view of the end-users about assistive technology, including AI, the needs of the end-users during their daily life activities, and the features that end-users expect technologies such as MAIA would have. Findings will fulfill the MAIA project by informing MAIA researchers and developers about the needs of the post-stroke population, their opinions about the assistive technologies, whether and to what extent AI could be useful for post-stroke circumstances, and participants’ concerns about the system.

## 2. Methods

### 2.1. Participants and recruitment

Sixteen participants, 12 post-stroke patients, and four caregivers of a post-stroke patient (11 female and five males, *M_age_* = 57.19, *SD_age_* = 15.70) were either recruited by physicians of IRCCS—Istituto delle Scienze Neurologiche, Bologna, Italy or from a local stroke patients’ association (i.e., A.Li.Ce, Bologna, Italy). The exclusion criteria for the patients were: (1) severe psychiatric disorders (i.e., psychosis), and the state assessment test (4AT, www.the4AT.com) was administered in case of doubt, (2) behavioral disorders (i.e., severe psychomotor agitation), (3) cognitive disorders or a state of confusion defined by temporal and/or spatial disorientation, (4) aphasic disorder or severe deafness, language comprehension skills below 75% in an ordinary conversation due to aphasic disorder or severe deafness (despite hearing aid). The token test was administered before the recruitment in case of doubt. Verbal expression ability below 75% in an ordinary conversation, even with facilitation by the caregiver. A simple oral fluency test (verbal fluency by phonemic category) was administered before enrolment in case of doubt, (5) inability to participate in videoconferences due to unviable technical requirements, (6) lack of support from a family member or friend if barriers due to technical knowledge or motor disability prevent the participant from participating in the video conferences despite having the necessary technical requirements (study protocol) (32). When the patient was excluded from the study due to the inability to lead the interview, his/her caregiver was recruited after firming the consent. The exclusion criteria for the caregivers were: (1) to be less than 18 and more than 80 years old, and (2) to respect points 5 and 6 reported for patients above.

Written informed consent was obtained before participation in the study. After the signed consent, the researcher assigned each participant a case report form (CRF) to guarantee anonymity. Once researchers obtained a list of selected participants, they called them to set up the interview details. Recruitment of participants was concluded when no new themes emerged over three consecutive interviews, which signifies the attainment of saturation in qualitative research terms (33).

### 2.2. Ethics

The Local Ethics Committees approved the project protocol (ASL_BO n. 0031849 provided on 29/03/2021; UNIBO n. 284787 provided on 05/11/2021) before the recruitment. The study was conducted according to the principles of the Helsinki Declaration. Participants were asked to sign written consent forms for participation and personal data handling and management.

### 2.3. Procedure

A socio-demographic questionnaire collected information about age, gender, highest education level attained, and years from injury. Then, semi-structured interviews were conducted to generate ideas, opinions, and constructive debate (34).

**Figure 1:**
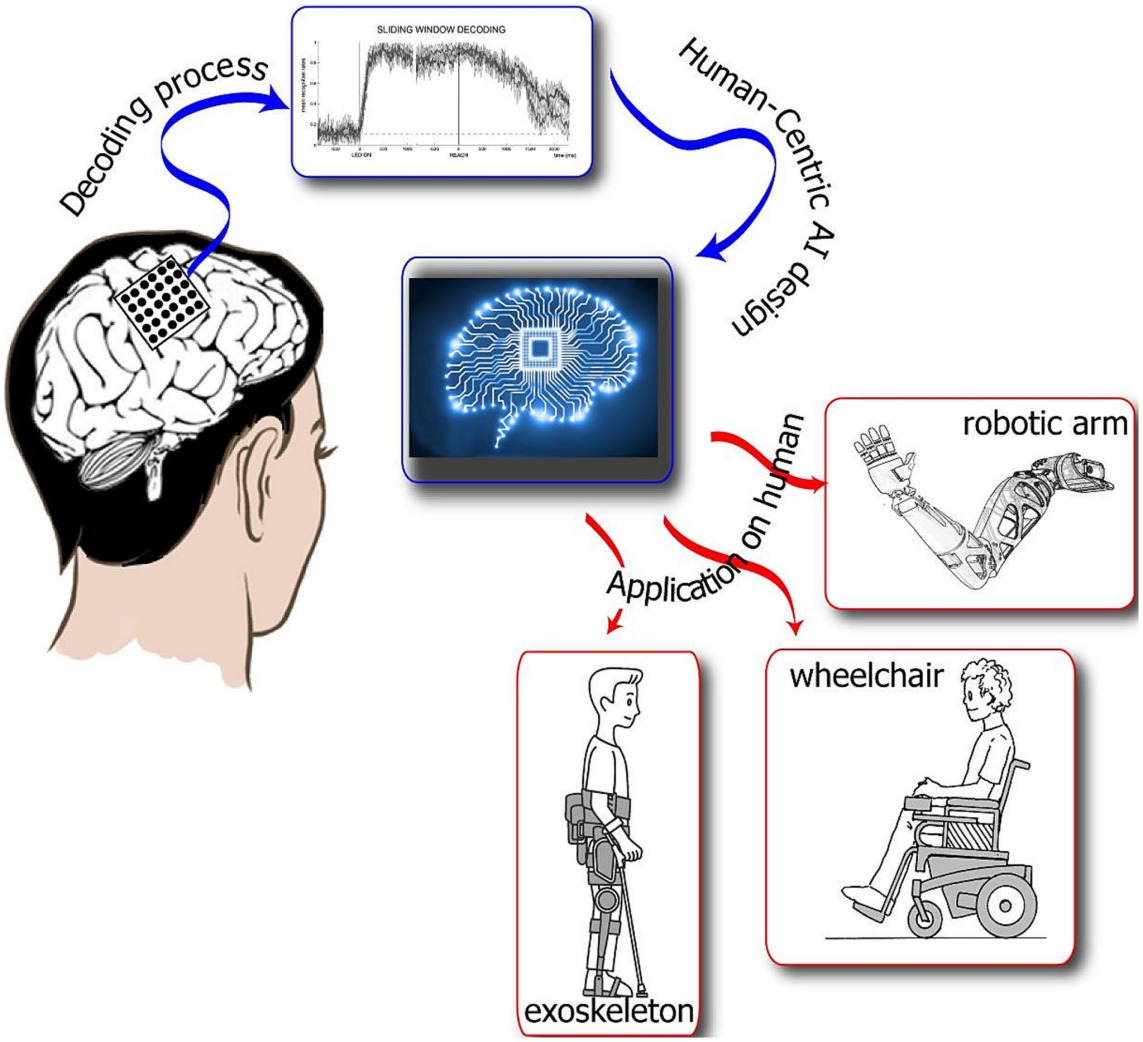
This is a visual presentation of the MAIA artificially intelligent system. MAIA technology will decode human intentions and communicate the decoded goals/targets to assistive devices and the users themselves to ensure compliance and develop trust through natural interaction and mutual learning. MAIA will combine sensorimotor inputs recorded from the posterior parietal cortex with data regarding eye movements to provide end-users with the control of robotic devices. Furthermore, MAIA will interact and have continuous bidirectional exchanges among multiple sensorimotor information to efficiently control the neuroprosthesis according to the end user’s intention and real needs.

Interviews were carried out by a researcher with experience in qualitative data collection (S.V.) and led online due to the COVID-19 pandemic. Each interview lasted between 30 and 45 min and was divided into three parts. During the first part, participants were encouraged to describe their motor abilities, previous or actual experiences with assistive technologies supporting them in daily activities, the impact of the motor limitations on their lives, and the support and care they received. Then, for the second time after recruitment, the researcher shared a PowerPoint presentation of MAIA technology describing all its features (Figure 1). During the last part of the interview, participants were invited to provide insights and feedback about MAIA and the characteristics the device should have to respond to their needs. In particular, they were asked to express their opinion on the best tool they could envision to communicate with the device (i.e., eye movements, voice, sound, and keyboards) and what role MAIA could have in their life. Finally, the interviewer asked participants to add details or clarify their answers when necessary.

### 2.4. Data processing

Three researchers performed an inductive thematic analysis (35) on the texts (SV, GO, and AT). The recorded audio tracks were transcribed verbatim and validated before the analysis. This analysis consisted of first gaining familiarity with the transcripts through repeated readings of the interviews. Then, the relevant codes representing the central units of content were extracted. Next, codes about the same issue were clustered into subthemes. At last, the results were discussed among researchers until a consensus about consistency was reached and any discrepancies solved (36).

The anonymized data are available at the following URL: http://amsacta.unibo.it/id/eprint/6854.

## 3. Results

### 3.1. Sample description

Descriptive statistics about the participants’ sample are presented in Table 1.

**Table 1:** Participants’ characteristics.

	Patient	Caregiver
Educational level		
High school diploma	9.3%	-
Bachelor degree	-	25%
Master degree	91.7%	75%
Years from injury		
0–2 years	58.3%	-
3–5 years	16.7%	75%^*^
>5 years	25%	25%^*^
Walking ability		
Yes, with difficulty, but without the need for aids	16.7%	-
Yes, with difficulty and need for aids (e.g., crutches)	83.3%	100%^*^
Aphasia level		
None	75%	-
Mild	25%	-
Severe	-	100%^*^
Autonomy level		
The patient eats alone with the dominant hand, and is independent in personal hygiene	83.3%	50%^*^
The patient eats alone with the dominant hand, but uses a male external catheter	16.7%	50%^*^

^*^data refers to the care-recipients (i.e., the patients).

### 3.2. Qualitative results

The thematic analysis identified three themes across the interviews (Table 2): (1) Needs to be satisfied; (2) MAIA Technology Acceptance; and (3) Perceived trustfulness. Each theme is described below, with examples extracted from verbatim of participants’ interviews.

**Table 2:** Theme and sub-theme from the interviews.

Themes	Sub-themes
1. Needs	Types of limitations
	Expectations
2. Technology acceptance	Perceived easy to use
	Perceived usefulness
	Adaption to new life conditions after a stroke
3. Trustfulness	Being alone with the technology
	Compliance with a brain implant

#### 3.2.1. Theme 1: needs

##### 3.2.1.1. Sub-theme 1.1: type of limitations

Walking, balance abilities, and mobility represented the primary needs emerging after a stroke and were indicated by the patients or their caregivers. However, when the lower limbs were less affected, and the patient could still walk (even with mild difficulty), the needs concerned mainly the upper limbs. In particular, the needs focused on regaining previous abilities: the need was more substantial when the damage interested the dominant hand and when the person used to do handicraft work or hobbies. Moreover, personal hygiene was another critical concern, as patients need constant caregiver assistance.

*My dream is to walk again; I miss the autonomy to walk. I have started to walk down the stairs but with enormous difficulty (Male patient).*

*My hobby was model building, taking old and broken objects, painting them, and putting them back together. Unfortunately, after the stroke, manipulating objects is impossible (Male patient).*

*An essential factor in her physical condition is her lack of balance; my wife is not free to go outside and constantly needs me (Caregiver, husband).*

##### 3.2.1.2. Sub-theme 1.2: expectations

Patients and caregivers showed interest in technologies like MAIA. Their primary expectation concerns regaining the lost autonomy because of their motor deficits. However, the expected technology supports were either rehabilitative or assistive. During the interview, participants declared their interest in exergames to rehabilitate cognitive abilities (e.g., attention and memory), in exoskeletons to train the lower limbs’ lost capabilities, or in technological gloves to stimulate the upper limb muscles. The rehabilitative technology included one designed to bring the lost abilities the most possible similar to the one pre-stroke, a possibility that occurs in a temporal window close to the stroke event. On the other hand, assistive technology includes the technology designed to act in place of the dysfunctional capability: this technology is deployed when no functional recovery is possible a few years after the stroke. Caregiver demonstrated interest in social robots capable of moving the care-recipient from one place to another.

*In my mother's case, we need technology to make her reasoning because she often forgets things and does not try to think to reason… she is childish (Caregiver, daughter).*

*I want a robot, like the ones you see in the movie, to help me to lift my husband from the bed and sit him on a wheelchair. Indeed, he is heavy, and I cannot do it alone (Caregiver, wife).*

*I want a system that sends the signal from my brain to my hand and allows me to open and close it (Male patient).*

#### 3.2.2. Theme 2: technology acceptance

##### 3.2.2.1. Sub-theme 2.1: perceived ease-to-use

Participants and caregivers were worried that the MAIA system might need effort. However, as participants explained, such an expectation derived from their need for technological skills. Notwithstanding the presentation of MAIA shown before the interview,^1^ participants were critical about its usability: patients worried that they would need the constant presence of some informal or formal caregivers, as when they have to use it for doing motor rehabilitation. On the other hand, the caregivers manifested the reciprocal feeling: based on their experience, their loved ones were incapable of using high-tech devices, such as smartphones and computers, and they did not feel MAIA to be so easy to use for their relatives. MAIA ergonomy was another aspect that emerged: patients and caregivers hoped MAIA would be as small as possible. Indeed, the participants’ houses were small and unsuitable for bulky technologies.

*The technology should be very light; the device you showed us seems bulky. Remember that we do not live in a castle; they must be designed for regular houses (Female patient).*

*I would always need the physiotherapist by my side because he only knows my condition, and I cannot lead a rehabilitation by myself (Female patient).*

*When developers create such technologies, they have in mind end-users people with easy learning. However, older people would not be willing; they would not be able to use this technology (Male caregiver, son)*.

##### 3.2.2.2. Sub-theme 2.2: perceived usefulness

Patients and caregivers believed that MAIA could enhance their motor abilities only if it would embody a rehabilitative technology, such as a glove for hand stimulation or an exoskeleton to train the mobility of the lower limbs. Most patients stated that technology for the upper limbs is unnecessary because they can use the non-dominant hand to fulfill their needs. Furthermore, participants believed that MAIA would fit better with people with severe motor injuries, particularly those who cannot move.

*The technology should prioritize more walking ability over arm activity because, with my arms, I can do most of my daily life activities (Female patient).*

*Fortunately, my mother does not need MAIA because she can regain her lost motor abilities after the rehabilitation (Female caregiver, daughter).*

*I think MAIA is more suitable for people with severe disabilities. Fortunately, I have achieved reasonable autonomy, but MAIA could help to rehabilitate my arm further (Female patient).*

##### 3.2.2.3. Sub-theme 2.3: adaptation to new life conditions after a stroke

Participants reported that after physical rehabilitation and once motor abilities were stabilized, they learned new strategies to adapt to their new physical condition and life. In this light, an assistive device might be meaningless to enhance daily activities.

*MAIA is an exciting technology, but I need to learn how to integrate it into my life because, by now, I have gotten used to doing everything with my right arm (Female patients).*

*My husband does everything with his right hand, which is dominant, and he has abandoned using the left hand (Female caregiver, wife).*

#### 3.2.3. Theme 3: perceived trustfulness

##### 3.2.3.1. Sub-theme 3.1: being alone with technology

Multifunctional Adaptive and Interactive Artificial Intelligent system evoked different feelings of trustfulness as a function of the point of view. Caregivers felt they needed to be more confident about leaving their care-recipient alone while using MAIA. Indeed, patients with cognitive impairments, including attention deficit and low-risk perception, need more assiduous assistance. For example, when a patient drives an electronic wheelchair, caregivers are used to staying by the patient’s side to keep control of their loved one crossing the street without checking the car coming. On the other hand, patients demonstrated to be highly confident in MAIA and believed to have the strength to control all the eventualities.

*I do not trust to leave my husband alone with the technology. I have to stay by his side constantly (Female caregiver, wife).*

*I can stay alone if I do not take risks and the technology is safe (Male patient).*

##### 3.2.3.2. Sub-theme 3.2: compliance with a brain implant

The implant of the brain sensor scared most patients and caregivers. They were skeptical because several had already experienced head surgery (e.g., for hemorrhage drainage or hydrocephalus) and did not want to undergo surgery again. In addition, pain represented another obstacle: none wanted to suffer for surgical interventions not assumed to be lifesavers. On the caregivers’ side, they were afraid of the extra caring work they could be required after the implant and the post-surgery extra costs, even in terms of money and time.

Differently, should the implant guarantee patients the relief of motion capabilities and symptoms, the trade-off between cost and benefit could be re-evaluated. In this light, two participants with previous AT experience and technological knowledge expressed their will to undergo the implant because they reported having nothing to lose.

*I consider the surgery very dangerous; she already had several brain surgeries for the drain valve, and I know what it means. It is also a psychological and emotional effort because it is not easy to recover after surgery, and I do not want to live again in that period (Male caregiver, husband).*

*I am willing to have surgery. The technology was built to help me, so I am not afraid (Male patients).*

*If there are no other ways to regain autonomy, the doctors guarantee that I will walk again and that the surgery is free of risks, then I agree with the implant (Female patient).*

*To regain motor autonomy, I would do anything, even right now (Male patients).*

## 4. Discussion

The present study investigated the features an assistive AI technology (i.e., MAIA) should embody to respond to stroke survivors’ and caregivers’ daily needs (32). It would represent an ambitious human-augmented technology for the loss of abilities due to stroke. Specifically, by capitalizing on the data of this study, the AI could finally be capable of decoding human intentions. Moreover, via natural interaction and mutual learning between itself and each user, the AI should become capable of translating intentions into actual actions. The approach the study adopted was human-centric, implying that the development of AI technology is grounded on analyzing potential users’ needs (37). In fact, when designing augmentation technology for restoring capabilities, user experience and social acceptability should be considered carefully because they affect people’s willingness to utilize the technology (22). Unfortunately, almost 75% of assistive technologies are abandoned by patients and caregivers when their needs and involvement are not considered during the technology production processes (28). Hence, to guarantee that MAIA may represent a suitable solution, we interviewed stroke patients and caregivers to ensure that potential users’ actual needs and expectations are considered as close as possible, besides avoiding an early drop-out.

From the conducted interviews, three primary themes surfaced: (1) needs, (2) acceptance of technology, and (3) trustfulness. The “needs” theme highlighted that the technology should empower patients to overcome their limitations. Participants indicated the necessity for the technology to compensate for lost abilities, depending on whether their impairments affected their upper or lower limbs. Notably, the loss of mobility due to lower limb deficits emerged as a particularly profound challenge, contributing to feelings of isolation among patients. These findings validate a comprehensive review of unaddressed, prolonged needs following strokes (38). This review emphasized that common unmet needs included issues related to mobility (identified by 46% of respondents), engagement in hobbies and leisure activities (as reported by 64.4% of respondents), and resuming employment or paid work (mentioned by 59.6% of respondents) (38). These unmet needs can potentially act as risk factors for patients’ mental well-being, given that the absence of work and leisure activities could heighten the likelihood of post-stroke depression. Consequently, this could further exacerbate the functional status and overall quality of life for both patients and caregivers (39).

When the primary deficit interested upper limbs, patients expressed the need to become capable of performing actions with their upper limbs again. Such a need was more salient when patients were involved in handcraft activities such as bricolage, gardening, baking, or writing, all requiring fine object manipulation, before the stroke. This aspect is related to the role of self-efficacy, namely the belief in one’s capabilities to organize and execute the courses of action required to produce given attainments (40). A previous study found the modulating role of self-efficacy on a patient’s quality of life and functional independence, as losing the ability to do specific activities enhances depression (41). In this perspective, assistive technology may play a key role in improving the patient’s quality of life as it may substitute for hand-lost abilities.

Furthermore, our results demonstrated that the motivation to regain upper limb motor abilities was higher in socially active patients until the stroke event, such as those who have not retired yet (42, 43). This finding aligns with the published literature that found lower life satisfaction in occupational stroke survivors than those who retired (44). Therefore, a possible explanation is that retired patients may be less unhappy and maintain a social identity despite their job, based not only on age and social characteristics but also on an individual’s sense of self (45).

Regarding restoring walking ability and/or hand motor functions, as mentioned above, patients from the acute phase to the first 2 years of injury required rehabilitative technology, such as a lower limb exoskeleton to regain walking abilities or a technological glove to rehab the hand grasp ability. A significant number of patients in our study retained optimistic prospects regarding their recovery, mainly due to the fact that they were in the early stages of post-stroke, within a 2-year timeframe. A noteworthy portion among them maintained hopeful aspirations for a complete restoration of their motor capabilities. On the other hand, after 2 years post-injury, patients were more interested in assistive technology, such as a lower limb exoskeleton. In particular, they aimed for technology to assist them in body balance, walking activities, or a robotic arm to reach and manipulate objects. This data align with the literature: hope for motor recovery decreases as the years from injury increase (46).

As regard caregivers, they look for assistive technology to help themselves (in assisting the patient) in activities they cannot do physically, such as moving the patient or helping the patient with personal hygiene. In this regard, technological development is moving toward robot-assistive living, which could be an optimal solution for the caregiver’s needs (47). The caregiving role is a potential stressor that might lead to various adverse health and well-being outcomes, including strain, burden, and depression (20). A potential resolution proposed by caregivers involves the implementation of social robots. The escalating demand for rehabilitation and aid, coupled with the increasing population of informal caregivers, have spurred innovators to create novel robotic systems that can seamlessly integrate into patient care solutions. Initial outcomes concerning social robots have demonstrated their tangible benefits, as they aid users in managing day-to-day activities and addressing age-related challenges (47). The popularity of social assistance robots is on the rise, encompassing their utilization in both senior care institutions and domestic settings. Additionally, recent research has unearthed that senior individuals are open to forming connections with robots in care facilities, viewing them as companions or familiar entities, with a substantial level of acceptance for the technology (48).

The second theme that emerged from the thematic analysis regarded technology acceptance, namely the characteristic that MAIA should have to align with the participants’ needs. To this end, patients and caregivers stated that MAIA has to be easy to use regarding technical aspects. According to Davis (27), the perceived ease of use is the degree to which a person believes that using a particular system would be free from effort. In our study, participants lacked technological skills and feared needing regular customer care service. This fact is relevant to consider when developing technology: developers should take care of the potential user’s tech abilities to avoid manufacturing potentially useless devices (49).

Moreover, in addition to its ease of use, participants emphasized the significance of MAIA’s usefulness, which essentially refers to whether they perceived it as a valuable tool for their intended purposes (27). For instance, if a patient has adapted to eating with their healthy hand and can manage without the injured limb, an arm robot might appear impractical and unnecessary for their needs. Regarding this aspect, we figured out an essential social aspect after a stroke: patients have become accustomed to living with their new motor abilities, such as doing daily activities with a healthy hand, walking, or moving by wheelchair or walker. Consequently, the perceived usefulness of MAIA could have been higher. This result is not surprising, and literature has already identified the coping strategies, namely the set of cognitive-behavioral strategies used by patients to manage the internal-external demands of the new life after a stroke, and the crucial role in doing activities without any more assistance than they usually use (50).

Another important aspect that emerged from the analysis was trustfulness toward the technology. More recent approaches have added the concept of trust as a predictor of technology acceptance (51–53). Trust can predict the reliance on technology, so low trust in skilfull technology would lead to disuse and high costs in terms of lost time and work efficiency (54). This study revealed divergent levels of trust between patients and caregivers. Patients tended to overestimate their confidence in promising technologies, possibly due to their deficits in attention and risk assessment (55). Alternatively, their inclination could stem from a willingness to embrace any potential solution that could enhance their physical and social circumstances or offer a glimmer of hope. Conversely, caregivers were concerned about the care recipient’s ability to interact with technology. Consequently, they were reluctant to accept MAIA as an assistive or rehabilitative tool and were hesitant to leave the patient alone with it.

One of the main characteristics of MAIA is the implantation of a sensor in the patient’s brain to allow AI to analyze action intentions from the parietal cortex and translate them into action to perform via the device. Regarding this aspect, both patients and caregivers were critical with respect to brain surgery. Indeed, most of our patients had already undergone head surgery and, as such, were refractory to new surgical interventions requiring hospitalization and its consequences. Furthermore, concerning compliance with the implant, the issue of the cost–benefit relation emerged: patients who wanted to return to work or were not retired at the stroke manifested interest in the brain implant. It is worth emphasizing that MAIA is presently a proof of concept, and participants were only introduced to it through an online presentation, lacking a concrete understanding of its potential. This limited exposure resulted in a lower level of trust. However, we expect that as participants gain access to the beta version of MAIA and can engage with it first-hand, their level of trust is likely to increase. This, in turn, could shift the cost–benefit relationship to a more favorable stance.

## 5. Conclusion

Stroke is the leading cause of motor disability with a substantial limitation on the patients’ and caregivers’ autonomy. The growth of technology and the potential of AI devices have opened the door to new solutions of human-augmented technology to restore patients’ autonomy and improve their quality of life after a stroke. To this end, the user-centric approach to co-design the MAIA technology (32) resulted in a productive method to build a solution as close as possible to the needs of the end users and avoid further technological abandonment. To build MAIA, developers should consider the technical abilities of the stroke population, asking for a device that is light, ergonomic, easy to use, and with barely invasive technology.

The forthcoming phases of the current study involve an examination of the acceptance levels concerning the beta iteration of MAIA. The upcoming investigation into user experience will involve the incorporation of focus groups and quantitative metrics to gain a more comprehensive understanding of MAIA’s usability. On the one hand, utilizing questionnaires to probe into technology acceptance and attitudes toward technology can provide valuable insights into individuals’ inclinations to embrace potential tools. On the other hand, conducting focus group discussions among individuals who share a similar post-stroke condition could serve as an ideal setting for gathering significant insights that contribute to the advancement of beneficial technologies like MAIA. Regrettably, due to the challenges posed by the COVID-19 pandemic, carrying out focus groups with post-stroke patients was hindered by their delicate health conditions.

## Data availability statement

The datasets presented in this study can be found in online repositories. The names of the repository/repositories and accession number(s) can be found at: https://doi.org/10.6092/unibo/amsacta/6854.

## Ethics statement

The studies involving humans were approved by ASL_BO n. 0031849 provided on 29/03/2021; UNIBO n. 284787 provided on 05/11/2021. The studies were conducted in accordance with the local legislation and institutional requirements. The participants provided their written informed consent to participate in this study.

## Author contributions

GO, RC, RP, FL, and AT made substantial contributions to the conceptualization of the project. SV, GO, GL, EM, FL, and AT made substantial contributions to the formal analyses, data collection, and drafting of the manuscript. SV, GO, GL, RC, LS, EM, RP, FL, and AT provided final approval of the version to be published and agreed to be accountable for all aspects of the work by ensuring that questions related to the accuracy or integrity of any part of the work are appropriately investigated and resolved. All authors contributed to the article and approved the submitted version.

## Funding

This work was supported by grant H2020-EIC-FETPROACT-2019-951910-MAIA, funding from the European Union’s Horizon 2020 research and innovation program under grant agreement No 951910.

## Conflict of interest

The authors declare that the research was conducted in the absence of any commercial or financial relationships that could be construed as a potential conflict of interest.

## Publisher’s note

All claims expressed in this article are solely those of the authors and do not necessarily represent those of their affiliated organizations, or those of the publisher, the editors and the reviewers. Any product that may be evaluated in this article, or claim that may be made by its manufacturer, is not guaranteed or endorsed by the publisher.

## Footnotes

^1^ https://site.unibo.it/maia-fetproact/en/for-patients-and-caregiver
